# Review 1: Lung transplant—from donor selection to graft preparation 

**DOI:** 10.1007/s00540-020-02800-z

**Published:** 2020-05-31

**Authors:** Zhaosheng Jin, Zac Hana, Azeem Alam, Shamala Rajalingam, Mayavan Abayalingam, Zhiping Wang, Daqing Ma

**Affiliations:** 1 Division of Anaesthetics, Pain Medicine and Intensive Care, Department of Surgery and Cancer, Faculty of Medicine, Imperial College London, Chelsea and Westminster Hospital, London, SW10 9NH UK; 2 Department of Anesthesiology, Affiliated Hospital of Xuzhou Medical University, Xuzhou, Jiangsu China

**Keywords:** Donor selection, Graft rejection, Lung transplantation, Postoperative complications

## Abstract

For various end-stage lung diseases, lung transplantation remains one of the only viable treatment options. While the demand for lung transplantation has steadily risen over the last few decades, the availability of donor grafts is limited, which have resulted in progressively longer waiting lists. In the early years of lung transplantation, only the ‘ideal’ donor grafts are considered for transplantation. Due to the donor shortages, there is ongoing discussion about the safe use of ‘suboptimal’ grafts to expand the donor pool. In this review, we will discuss the considerations around donor selection, donor-recipient matching, graft preparation and graft optimisation.

## Introduction

Solid organ transplant began in the 1950s with a kidney being the first successful organ to be transplanted. In 1963, Dr James Hardy performed the first recorded lung transplantation, but the patient died after 18 days postoperatively. Over the next 2 decades, a number of additional attempts were made, all with early postoperative mortality. In 1981, Dr Norman Shumway and Dr Bruce Reitz performed the first successful heart–lung transplants on three patients, two of whom survived. Two years later, Toronto Lung Transplant Group performed the first successful single lung transplant and the patient survived for 8 years postoperatively [1].

Fast forward to the present, the International society for Heart and Lung Transplantation (ISHLT) has recorded over 64,000 cases of adult lung transplants. The median survival after lung transplant has also increased from 4.3 years in the 1990s, to 6.5 years in the last decade [2].

Indications for lung transplant are due to end-stage lung disease and can be sub-categorised into: Obstructive disease, e.g. Chronic Obstructive Pulmonary Disease (COPD); Fibrotic disease, e.g. Interstitial pulmonary fibrosis; Septic disease, e.g. Cystic Fibrosis, and Vascular disease, e.g. Pulmonary hypertension [3]. ISHLT report in 2016 indicates that COPD is the most frequent indication for lung transplant, accounts for 31% of the surgeries undertaken from Jan 1995 to June 2016, followed by 24.9% for interstitial pulmonary fibrosis and 15% for cystic fibrosis [4].

This review aims to update our current understanding regarding a few key elements of pre-transplant consideration, such as donor selection, importance of ischaemic time, and the methods of graft preservation during storage.

## Lung transplant around the world

According to data collected by Global Observatory on Donation and Transplantation (GODT) on behalf of the world health organization (WHO), there were over 5500 transplants carried out in 2017. Over 3,000 lung transplants were carried out in America, 2200 in Europe, 231 in western Pacific, 37 in Mediterranean. Africa last reported 14 cases of lung transplants in 2016, while the last reported case from South East Asia was in 2015 [5]. When divided by countries, United States carries out by far the highest number of lung transplant per year, with 2449 operations in 2017 [6]. In Europe, Germany, France and Spain each carried out over 300 cases according to data from previous years; while United Kingdom reported 207 cases in 2017 [6, 7]. Other countries with significant lung transplant activities includes Australia, Japan and China [7–9]. The proportion of circulatory death donors (DCD), in European countries and Australia are fairly consistent, at 20–25% [10, 11]. Interestingly, an US report suggests that only 4% of the lung transplants in US are from DCD donors [12].

According to the ISHLT 2017 report, 1-year survival after lung transplant world-wide is reported at 84%, while 5-year survival is 57% [2]. From the data available from various sources, 1-year survival in US is reported to be between 80–90% in the US, Europe and Australia [6, 13]. Five year survival is reported to be over 70% in Japan and Australia [10, 14], compared to 55–57% in the US and UK [6, 15].

## Donor selection

**Fig. 1:**
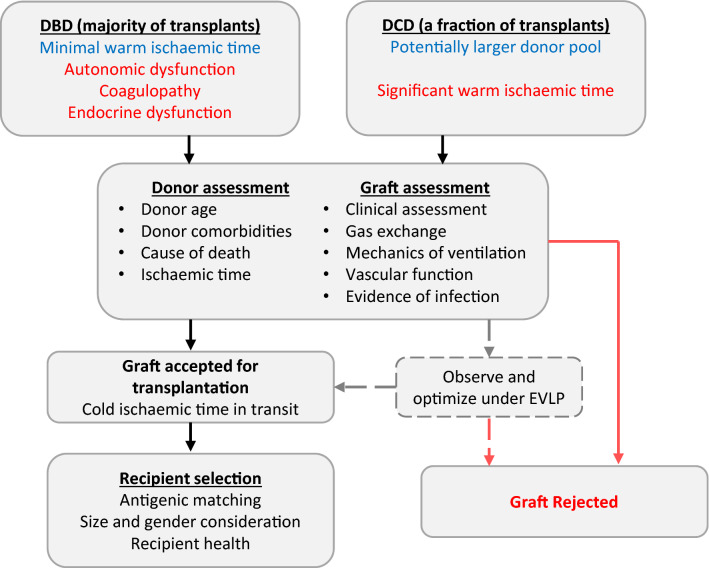
Process of graft and recipient selection, *DBD* donation after brainstem death, *DCD* donation after circulatory death, *EVLP* Ex Vivo Lung Perfusion

In the early years of lung transplant, the selection process for lung graft donor was highly stringent. Criteria included age under 55 years, less than 20 pack-year smoking history, no history of pulmonary disease, absence of systemic or pulmonary infection, normal gas exchange and clear chest radiograph (Fig. 1). As a result, most of the lung grafts was retrieved from patients with traumatic head injury and brainstem death [16].

However, as lung transplant has now become one of the mainstay treatments for end-stage lung disease, availability of organ has become a major limiting factor in transplant surgery. As of March 2018, there were 353 patients on the active UK lung transplant wait list but only 207 lung transplants performed in the 2017/2018 financial year [15]. 25% die within 2 years of being listed on the UK lung transplant list [15]. The data in the US are similar, with 1462 patients on the lung transplant waiting list [17].

The shortage of donors, as well as the increasing clinical experience on the post-transplant care has led to ongoing discussion regarding the balance between the outcomes of utilizing suboptimal lung grafts and the mortality while on the waiting list. In this section, we will discuss the main donor considerations (Fig. 1).

### Donor age

Even in absence of pulmonary pathology, aging is associated with loss of alveolar surface area [18], as well as reduced alveolar gas exchange [19, 20]. In addition, aging is also associated with the loss of connective tissue content of the lung, which results in the gradual decline in the elastic recoil and impairs alveolar emptying during expiration [21]. This is demonstrated as increase in functional residual capacity (FRC) with age [22]. In addition, the loss of connective tissue also weakens the structural support of the small airways, making them more prone to collapse during expiration. According to Laplace’s law, collapsed airway requires significantly more pressure to expand, therefore, increasing the work of respiration. Indeed, it is thought that in patients over 60 years of age, closing capacity (the lung volume at which alveolar and small airway begins to collapse) becomes higher than the FRC, meaning the collapsed areas need to be re-expanded after each breath, leading to significantly higher work of respiration [23].

The aging process also impairs the immune function of the respiratory system. Studies have demonstrated that mucocillary clearance time is significantly longer in the elderly, this is due to reduced ciliary beat frequency and ultrastructure [24]. Immune cells that line the alveolar surface and conducting airways form part of the innate immune system and are important in lung antimicrobial defences [25]. The functions of these cells change with age and may affect underlying processes in primary and chronic graft dysfunction as well as its ability to clear infections [26].

It can be summarised that even in absence of any other lung pathology, lung graft from older donors are likely to have reduced ‘physiological reserve’ for gas exchange and minute ventilation. This coupled with the increased risk of infection is likely to lead to worse outcomes after transplant. Indeed, the latest data set of the ISHLT registry showed donor age to be a statistically significant risk factor for 1, 5 and 10 year mortality, thus making grafts from older donors less favourable (Fig. 1) [2]. On the hand, Katsnelson et al. categorised 3227 elderly patients aged 65–80 years receiving their first lung transplant into 2 groups; donors ≥ 10 years younger than recipients and donors within 10 years of age of recipients. 263 donors (8.15%) were within 10 years of their recipient’s age at transplantation. There was no difference in overall or intermediate conditional survival past 1 year between groups [27]. The reason for this may be the larger data set included in the ISHLT analysis (over 30,000 patients in the ISHLT analysis vs 3227 in Katsnelson’s report). The increased susceptibility to infection and poor functional reserve of older lungs would need to be balanced with waiting list mortality in decisions regarding accepting lung grafts from older donors [26].

### Comorbidity and other donor characteristics

In addition to patient age, the ISHLT report also identified a number of donor comorbidities as significant risk factors for post-transplant mortality; this includes donor smoking history, diabetes and donor cytomegalovirus (CMV) infection (Fig. 1).

The association between donor smoking history and adverse events after transplant is not surprising. Smoking is associated with a wide range of pathological changes to the airway and lung parenchyma [28] and with deterioration in lung function [29]. In addition to the ISHLT data, several other studies have reported worse post-transplant outcomes in where donor had positive smoking history [30]. Interestingly, Sabashnikov et al. reported in their study that smoking history is not associated with significant difference in the immediate postoperative outcomes or survival up to 3 years postoperatively. It is worth mentioning, however, the above study contained a relatively small case number, which was divided into three subgroups (non-smokers, smokers, heavy smokers) [31].

Diabetes is a systemic disease with a multitude of end organ sequalae, which includes impairment of lung function. Similar to its effect on other organs, diabetes is thought to have deleterious effect on the pulmonary vasculature, leading to an increased risk of pulmonary vascular disease [32]. Population studies have reported that diabetes is associated with significantly worse lung mechanics, with lower FEV1/ PEFR and FVC [33–35]. In addition, diabetes is also associated with significantly worse alveolar gas exchange [35, 36]. Functionally, patients with diabetes were found to have significantly shorter 6-min walk distance [33]. The association between diabetes and prognosis after lung transplant has also been observed by Ambut et al., however, they further reported that the association was found only in single lung transplant surgeries, but not in double lung transplant [37]. The underlying mechanism is not clear, however, the authors attributed this to the graft characteristics difference between single and double lung transplant.

CMV is a DNA virus of the *Herpesviridae* family. In immunocompetent individuals, CMV infection commonly presents with pharyngitis or are completely asymptomatic. After the primary infection, the virus can establish lifelong sub-clinical infection known as latency, this can occur in several tissue and organs, including the lungs [38]. In transplant patients on immunosuppressants, CMV infection may result in several life-threatening complications, including CMV pneumonitis. The issue of post-transplant CMV infection will be discussed in detail in the review 3.

In addition to the comorbidities discussed above, the ISHLT report also identified several other donor and recipient characteristic mismatches with significant association to the postoperative outcomes, this included donor and recipient height, BMI and gender. Shorter donors (by more than 20 cm) but not shorter recipients are associated with significant 1-year mortality [2, 4]; underweight and obese donors, as well as donor–recipient BMI mismatches are all associated with worse post-transplant prognosis (Fig. 1) [39, 40]. Finally, the ISHLT 2018 reported indicated that donor and recipient gender combination plays a role in the post-transplant prognosis, with significantly worse mortality in gender mismatched transplants compared to female donor–female recipient transplants [41]. Demir et al. analysed 461 transplant cases in a single centre in Belgium, and further stratified gender pairing into high risk (female donor–male recipient), low risk (female donor–female recipient), and intermediate risk (male donor–male recipient and male donor–female recipient) (Fig. 1) [42]. However, the exact risk of each gender combination is not clear, as other studies have reported highest mortality rate in male donor–female recipient combination [43].

### Antigenic matching

In the early years of lung transplant, the consensus was that similar to blood transfusion, the donor and recipient must be ABO compatible. However, due to concerns over donor derived antibodies causing haemolysis and graft failure, some centres advocated the use of ABO-identical donor recipient combinations [44, 45]. However, since then a number of studies have reported no significant difference between long-term survival between ABO identical and ABO compatible transplants [42, 46]. Fakhro et al. published a comparative study of 297 lung transplant cases and reported that while 1-year survival was up to 7.5% higher in the ABO-identical donor recipient combination, the survival rate beyond 1 year was not significantly different between the cohorts. This, however, needs to be balanced with the consideration that the median waiting time for the transplant was almost doubled in the ABO-identical cohort [47].

With the advent of ABO incompatible renal transplants [48], the possibility of ABO incompatible lung transplants have been considered, either due to life threatening need for urgent transplantation or inadvertent transplant of incompatible grafts. Management principles involve immediate perioperative plasmapheresis and intravenous immunoglobulin administration, followed by long-term immunosuppression regime [49–52]. The longest reported survival case is a 17-year-old boy who had an inadvertent incompatible graft transplanted, who was followed-up and survived for 9 years, albeit with progressively deteriorating lung function [51].

Human leucocyte antigen (HLA) is a gene complex which contains the genes for numerous cell surface proteins responsible for presenting antigens and regulating the immune system [53]. These are inherited in a codominant fashion; therefore, any individual would have two sets of three HLA genes which needs to be considered. In cases where the donor and recipient does not have six identical HLA genes, the recipient immune system could adapt and mount an immune response against the protein of the unmatched HLA gene, resulting in graft injury and ultimately graft failure [54]. The importance of HLA matching has been reported in a number of large observational studies, including the 2017 ISHLT report, and a 23,000-patient study reported by Hayes et al. (Fig. 1) [2, 55]. It is worth noting that, while Hayes et al. employed a lower cut-off for HLA mismatch (< 3 vs ≥ 3), their study actually reported a lower hazard ratio for mortality compared to the ISHLT report, which suggests that having three HLA mismatch may not be associated with significantly worse outcome.

## Donor graft preparation and allograft ischaemia time

While there has been reports of living lung donation, deceased donors account for the vast majority of lung grafts. Deceased donors are categorised into DCD donors or donation after brainstem death donors (DBD). Currently, DCD donors account for approximately 20% of the lung transplant procedures worldwide. In this section, we will discuss the issues regarding lung graft from DCD and DBD donors, as well as the graft extraction process.

Ischaemia time represents the time between cessation of the donor circulation to the reperfusion of the graft in the recipient. This is divided into warm and cold ischaemia time. Warm ischaemia time refers to time with reduced organ perfusion starting before cardiac arrest to start of organ preservation and cooling with solutions [56]. It is generally accepted that lowering the graft temperature reduces metabolic requirement, which reduces the extent of the graft injury under ischaemic condition [57]. DCD lung allografts will usually have degree of warm ischaemia, as there will invariably be a time-window between inadequate organ perfusion and cessation of circulation. The process involves certain key time points which would benefit from standardisation to enable data comparison. Cypel et al. defined 5 time points starting with withdrawal of life sustaining treatment (T0), oxygen saturations < 80% (T1), systolic blood pressure < 50 mmHg (T2), cessation of cardiac output/asystole (T3), resumption of lung inflation/ventilation (T4), and start of pulmonary flush (T5) [58]. It is generally accepted that warm ischaemia should be limited to less than 60 min [59]. However, small studies have suggested that longer warm ischaemic time may not be associated with worse post-transplantation outcomes [60, 61]. If this holds true, however, and longer intervals from withdrawal of life sustaining treatments to arrest result in acceptable transplant outcomes, it may be possible to significantly expand the donor pool [58].

DBD lung allografts on the other hand have minimal warm ischaemic time as circulatory arrest is not required prior to organ procurement [2]. Therefore, ischaemic time in DBD is primarily cold ischaemia. The 2017 ISHLT report examining lung transplants between 2009 and 2015 and found that cases with more than 6 h cold ischaemia time was associated with significantly lower 30-day survival rate. However, the difference in survival is not seen in beyond 1 year follow-up [2, 4]. However, the report had a poor data completion rate with difficulty adjusting for potential confounders, hence long-term survival conclusions cannot be made yet without further research.

### Donation after brainstem death

Most organ transplantations in the UK are performed using heart beating brain-dead organ donors.

The process of brainstem death is often associated with a predictable pattern of complex multiple organ failure which can result in rapid deterioration in the function of the transplantable organ prior to retrieval, unless these pathophysiological processes are actively managed. Clinically, the central nervous system induces a triad of physiological changes described by Cushing; namely hypertension, bradycardia and apnoea in response to raised intracranial pressure [62]. Brainstem death results in a systemic pro-inflammatory environment mediated by cytokine release, which the body subsequently responds to by causing a surge in circulating catecholamine release, thus creating a concomitant ‘autonomic storm’ [63–66]. The cytokine and catecholamine burden on organ tissues and vasculature can precipitate the development of adverse inflammatory, haemodynamic and endocrine deleterious sequelae, including intense vasoconstriction, tachycardia, pulmonary oedema, myocardial damage, as well as both microvascular and parenchymal damage to distant organs, including the transplantable graft [66–71]. The period of profound autonomic activity is followed by a sudden drop in catecholamine release, which is associated with subsequent bradycardia, systemic vasodilation, and ultimately tissue hypoxia and necrosis [72]. These deleterious processes are accentuated by a profound coagulopathy, which is theorized to be partly due to release of tissue thromboplastin from the necrotic brain with the subsequent development of disseminated intravascular coagulation, which can have a significant effect on graft viability [73, 74].

Endocrine and metabolic changes have also been described. In humans, most commonly posterior pituitary function is lost and results in diabetes insipidus and the resultant fluid and electrolyte abnormalities, whilst anterior pituitary function may be preserved or minimally affected [74, 75]. It has been theorized that this may be due to preservation of pituitary perfusion [76]. Loss of hypothalamic function and central thermoregulation has also been observed, characterized by initial hyperpyrexia and subsequent hypothermia, whilst changes to thyroid function are characterized as ‘sick euthyroid syndrome’ [77, 78]. Furthermore, metabolic changes have also been observed, such as hyperglycaemia secondary to a reduction in insulin release, as well as insulin resistance [63, 64, 79].

Due to the pathophysiological changes described above, DBD organs are subject to a significant pro-inflammatory period associated with haemodynamic instability and autonomic dysfunction.

Finally, like all grafts, DBD organs experience a degree of subsequent ischaemia–reperfusion injury, which generates reactive oxygen species, activates the innate and adaptive immune systems and drives the release of cytokines resulting in inflammation, and potentially microvascular and parenchymal injury [80, 81].

To maximize recipient outcomes, it is imperative that patient monitoring, treatment goals and specific therapies are optimized peri-operatively. Whilst the precise methods by which to optimize graft outcomes remain to be elucidated, numerous principles of donor management have been suggested. These include, but are not limited to, ICU management with early correction of hypothermia [63, 64, 78], lung protective ventilation with a tidal volume of 6–8 ml kg^−1^ with optimal PEEP [82–84], close cardiac monitoring and inotrope support as required with active correction of hypovolaemia whilst avoiding overhydration and hypernatremia [71, 78, 85–88]. Furthermore, administration of a methylprednisolone bolus immediately after brain death has demonstrated improved utilization of heart and lung grafts [89, 90].

### Managing donor complications associated with donation after brainstem death

Brainstem death is associated with a predictable cascade of systemic inflammation and multiple organ failure. Optimal donor management during this period is vital to mitigate graft injury and ensure short- and long-term graft viability. Complications that require monitoring and management are varied, such as hypotension, hypovolaemia, coagulopathy.

Brainstem death is associated with a dramatic loss of sympathetic tone, resulting in profound systemic vasodilation and reduced cardiac contractility. Whilst the optimum management of this cardiovascular instability remains to be elucidated, it has been postulated that restoration of euvolemia is associated with improved postoperative graft function. A combination of crystalloids, colloids and blood components have been used to achieve this, and it has been suggested that sensible therapeutic objectives include maintaining a haemoglobin concentration above 10 g/dL and restoring intravascular volume, and colloid oncotic pressure [91].

Whilst the use of inotrope should be limited due to the risk of excessive vasoconstriction, as well as downregulation of adrenergic receptors, there is evidence to suggest that dopamine should be a first-choice inotrope [92]. In contrast, the use of noradrenaline may compromise graft viability. During this period, pulmonary artery catheter monitoring is often used to ascertain precise measurements such as cardiac output and pre-load, alongside optimising oxygen delivery. The aim is to maintain a mean arterial pressure > 60 mmHg.

Coagulopathy is a common complication of brainstem death and is thought to occur due to the release of plasminogen activator, thrombocytopenia and hypothermia [73]. Once again, there is no consensus as to the optimum management of this complication, however, a variety of blood products is often required and reasonable end points include a platelet account > 50,000 mm^3^ and an international normalised ration below 2.0.

A variety of other complications ensue following brainstem death, such as hypernatremia, hypokalaemia, and hypovolaemia secondary to diabetes insipidus [93], as well as arrhythmias [75], neurogenic pulmonary oedema [71], aspiration pneumonitis, hypothermia, and hyperglycaemia due to pancreatic insufficiency [94].

Various agents have been used in murine studies and in humans that have demonstrated some improvement in clinical outcomes, such as a combination of corticosteroids, vasopressin, insulin and thyroid hormone. Further investigation into novel cytoprotective regimens, such as the use of ischaemic preconditioning, anti-C5a and selectin inhibitors, remains ongoing.

These numerous clinical effects of DBD on donor physiology demonstrates the importance of improving clinicians’ understanding of the pathophysiology underlying these complications to optimise donor graft viability.

### Donation after circulatory death

DCD refers to the process of retrieving organs for transplantation that occurs following confirmation of death using circulatory criteria. The circulatory death refers to ceasing of brain perfusion [56]. Organs, therefore, are harvested from donors who have died or are awaiting cardiac death, rather than from brain-dead patients with a cardiac output, as in typical organ donation. DCD donors are generally patients who have been unsuccessfully resuscitated or are awaiting cardiac death. This generally encompasses patients that have suffered catastrophic brain injuries but do not fulfil the neurological criteria for death. However, despite this, these patients are experiencing significant injuries that would justify the withdrawal of life-supporting cardiorespiratory management in the patient’s best interests.

Due to the widening discrepancy between organ supply and demand, with demand continuing to increase, there has been a re-introduction of DCD donor schemes for various forms of transplantation, including organs with a low tolerance for warm ischaemia, such as the lungs, pancreas and liver. For example, Belgium, the Netherlands and the United Kingdom have effective DCD donor schemes, with an estimated 7.0–9.5 DCD donors per million population in 2013 [7]. This change in practice is particularly due to the finding that graft outcomes following selected DCD transplantations and DBD transplantations are similar [95–97].

The most widely used classification to categorise DCD is the modified Maastricht classification [98]. Category I describes patients who are dead on arrival to the hospital (therefore, not receiving cardiopulmonary resuscitation); Category II describes patients who have underwent unsuccessful resuscitation en-route to the hospital; Category III indicates awaiting cardiac or circulatory death, this is usually in cases of planned withdrawal of life-sustaining therapies; Category IV describes cardiac in a brain-dead donor (Table 1) [99]. Controlled DCD describes DCD which occurs after a planned withdraw of life-support, i.e. Category III; whereas Categories I, II and IV are generally thought to be uncontrolled.

**Table 1:** Maastricht classification of donation after circulatory death

Category I:	Patients pronounced dead prior to arrival at the hospital, cardiopulmonary resuscitation abandoned
Category II:	Patients with ongoing cardiopulmonary resuscitation on arrival, but unsuccessful
Category III:	Patients with planned withdrawal of life-sustaining therapies (controlled)
Category IV	Cardiac arrest after brain stem death

Compared to DBD grafts, DCD grafts sustain a greater degree of ischaemic insult prior to harvesting, when they are subsequently cooled and perfused. This is because, during DBD, organs undergo cold perfusion prior to organ harvesting, whilst in DBD grafts there is a definitive period between cardiac arrest and organ retrieval. This period is known as the “warm ischaemic time” and has been shown to affect organ quality. Studies have theorized that ischaemia impairs organ recovery due to stimulation of innate and adaptive immune responses, the generation of reactive oxygen species and the induction of apoptosis, resulting in hypoxic injury, inflammation and graft vascular disease [80, 81, 100–102]. In turn, these deleterious pathophysiological changes may increase the risk of delayed graft function (DGF), which has been shown to result in poor long-term graft function and patient survival [103, 104]. Due to the innate susceptibility of the kidneys to hypoxia, secondary to the organ’s significant metabolic demand, the long-term effects of ischaemia–reperfusion injury has been predominantly studied in the context of renal transplantation. Hypoxic injury has been shown to initiate kidney allograft dysfunction, acute reject; this reduces graft survival [96].

The most significant period of warm ischaemia occurs following the onset of asystole and the institution of cold perfusion, however, it is important to note that the initial period occurs as early as the preceding phase of cardiorespiratory deterioration. Once the procurement process has begun, extracorporeal membrane oxygenation (ECMO) is a critical technique that circulates blood to the transplantable organ, thereby limiting the duration of warm ischaemia. The period of warm ischaemia is higher in uncontrolled DCD donors, because by definition, the process of warm ischaemic injury has already been established by the time that the possibility for donation has been appreciated.

Overall, due to the prolonged warm ischaemic time observed in DCD donor transplantation, compared to DBD donation, the risk of ischaemic injury is significantly higher (Fig. 1). This may contribute to acute rejection, primary graft failure, delayed graft failure, as well as other ischaemic complications [105–109], such as biliary strictures [110]. As such, criteria for selecting viable DCD donors must be strict and adhered to, whilst limiting uncontrolled DCD donors and elderly DCD donors with co-morbidities such as hypertension, peripheral vascular disease and diabetes, as well as limiting warm ischaemic time with adjunctive therapies such as ECMO.

### Donation after circulatory death vs brainstem death

DCD rates started increasing in 2007 and now make up 17.8% of all lung transplants in 2017/2018 in the UK [15]. DCD is classified into Uncontrolled DCD (e.g. dead on arrival, patients with unsuccessful resuscitation post cardiac arrest) and Controlled DCD (inpatient withdrawal of life sustaining treatment and unexpected cardiac arrest in patients with known or suspected brain death) [56]. In the largest registry review comparing DCD (*n* = 306) and DBD (*n* = 3,992) lung allograft outcomes, no significant difference was found in 30 day (96% vs 97%), 1 year (DCD 89% vs DBD 88%, *p* = 0.59) or 5 year mortality (both groups 61%, *p* = 0.87) between the groups [58]. 94.8% of DCD donors in this review were Maastricht Category 3 where inpatient withdrawal of life sustaining treatment was performed [111]. Interestingly, early 30-day survival was significantly affected by donor mechanism of death with head trauma patients having worse early outcomes possibly due to silent micro-aspirations [58]. A meta-analysis of 6 observational cohort studies by Krutsinger et al. found no difference in 1-year mortality, acute cellular rejection and primary graft dysfunction between DCD and DBD allograft recipients [112].

In a retrospective single centre study evaluating transplants between 2007 and 2013, DCD lung transplant recipients (*n* = 59) were compared with DBD lung transplant recipients (*n* = 331) [61]. No significant difference was found between both groups with respect to primary graft dysfunction score (*p* = 0.67), chronic lung allograft dysfunction free survival (*p* = 0.86) and overall survival (*p* = 0.15) [61]. PGD was graded from 0–3 based on partial pressure of oxygen over fractional inspired oxygen concentration (Pa02/Fio2), chest X ray findings and need for ECMO at 72 h [61]. This occurred despite the significantly longer ischaemia time and significantly more donors with smoking history in the DCD group [61]. A prospective single centre study of 302 lung transplants of which 60 were from DCD donors showed no significant difference in acute rejection episodes (*p* = 0.98) and overall cumulative survival compared to DBD lung transplants after up to 7 years of follow-up [113]. However, the incidence of postoperative bronchiolitis obliterans syndrome (BOS) was significantly higher in DCD group (23.5%) than in DBD group (11.7%, *p* = 0.049) with significantly shorter BOS-free survival in the former (*p* = 0.028) [113].

In summary, while DBD organ grafts are often associated with shorter warm ischaemic time, the exposure to cytokines, catecholamine and haemodynamic compromise may still result in significant graft injury. Current data suggest that careful selection of DCD candidates may yield long term prognosis comparable to that of DBD candidates (Table 2).

**Table 2:** Summary of the characteristics of donation after brainstem death and donation after circulatory death

	Donation after brainstem death	Donation after circulatory death
Donation cohort	Patients that fulfil the criteria for brainstem death but maintain cardiac output	Donors who have died or are awaiting cardiac death
Proportion of donors	≈ 65%	≈ 35%
Warm ischaemic time	Minimal, due to maintenance of cardiac output	Usually prolonged, due to the interval after asystole where organs are not perfused and have not yet been cooled
Pathophysiological insult	Brainstem death results in systemic cytokine and cathecholamine release associated with haemodynamic instability and graft insult	Prolonged warm ischaemia stimulates the activation of innate and adaptive immune responses, generation of reactive oxygen species and induction of apoptosis
Graft outcomes	Current data suggest that careful selection of DCD candidates confers a long-term graft outcome that is comparable to DBD donors [60, 112]	

## Lung graft extraction and storage

### Preparation for transport

The lung graft is extracted through a midline sternotomy, followed by dissection of the graft, the airway and the vasculature. After the lung retrieval, a prostaglandin E1 vasodilator is given to reduce the pulmonary vascular resistance for a well distributed flush [114]. The graft is then prepared using a preservation solution with low potassium, dextran and glucose such as Perfadex^®^. It has been proposed that microthrombi formation after circulatory death could exacerbate graft injury, and studies have suggested that gas exchange, lung compliance and vascular resistance are better when the graft was flushed in retrograde fashion via the left atrium/pulmonary veins [115, 116]. The common practice in most transplant centre is, therefore, to carry out both antegrade and retrograde flushing [117, 118]. Once this process has been completed, the lungs are inflated with oxygen to maintain structure. Lung deflation and atelectasis while in storage has been suggested to worsen graft function, that inflation of the graft to 75–100% vital capacity, and with 40–60% O_2_ are associated with the best gas exchange and lung compliance [116, 119]. The lungs are then transported in one of two ways as described below.

### Cold storage method

Of the two methods, this is the more common. The concept is that lung grafts are flushed with buffered Perfadex^®^ and then preserved in a cold storage device for transport to their destination hospital. The lungs are cooled to prevent damage ex vivo before being gradually warmed for placement into the recipient. Cold storage is based around the concept that it slows metabolism, reduces oxygen consumption and substrate requirements thus preventing end organ deterioration [120].

Optimal temperature for storage appears to be between 4 and 8 °C. Cold storage has been suggested to compound certain aspects of ischaemia–reperfusion injury, through increased pulmonary vasoconstriction and thus increased vascular resistance after reperfusion [121].

### Ex vivo lung perfusion (EVLP)

EVLP systems provide normothermic, and continuous ex vivo perfusion of the lungs. This was initially designed for extracorporeal assessment and optimisation of the lung graft to increase the utilisation of ‘marginal’ grafts that would otherwise be rejected, thereby increasing the potential donor numbers [122, 123]. However, studies are also now looking into EVLP as a graft preservation strategy for transportation.

There are three main EVLP protocol currently in use, the Lund protocol, Toronto protocol and Organ Care System (OCS) protocol, with several key differences in operation. For example, with the Toronto protocol, the lung graft is perfused with an acellular Steen solution, and a funnel-shaped cannula is sewn into the left atrium, creating a closed circulation, whereas Lund and OCS protocol uses a mixture of packed red cells and Steen’s solution, with haematocrit between 15 and 20%, while the left atrium is left open with free drainage [124]. Most notably, OCS is currently the only portable EVLP system, which enables EVLP during transport, whereas the other two systems does not overcome the need for cold storage during transport [125]. In the next section, we will briefly describe the setup for OCS system.

The OCS system has various components which include a wireless monitor which displays and controls the system’s functions using specific parameters; lung perfusion module which acts like a casing for the lung within which the lungs are stored; a lung solution providing nutrients and essential contents for preservation of the lung outside the body optimizing for transplant [126]. The solution stored in the reservoir is pumped through the gas exchanger, then into the lung graft through the pulmonary artery catheter. The solution is then drained through the left atrium back into the reservoir, completing the circuit. As immune cell recruitment and activation are heavily implicated in the development of graft dysfunction, some EVLP systems have a leucocyte depletion filter which is thought to reduce inflammation and improve graft function [127]. At the same time, the lung is also ventilated using an integrated ventilator through a tracheal cannula [126]. An example of the protocol is described in the manufacturers manual, prior to connection to the lung graft, the system is firstly primed with the perfusion solution mix and warmed to 32 °C. The lung graft is trachea and the pulmonary artery cannulated and connected to the OCS. The graft is then gradually warmed up to 37 °C using low flow perfusion (manufacturer recommends 0.5 L per minute). At the same time, the lung is ventilated using volume-controlled ventilation mode at 6 mL/kg of donor’s ideal body weight, at a rate of 12 breaths per minute and positive end expiratory pressure (PEEP) of 5–7 cmH_2_O, and Fraction of inspired oxygen of 12%. This can then be maintained through the transfer process.

### EVLP and cold storage: effects on inflammation

Extensively invasive procedures, such as a lung transplantation, can be accompanied with ischaemia–reperfusion injury. Acute rejection, graft dysfunction, comorbities and mortality rates are affected by this ischaemia–reperfusion injury [128–130]. The underlying cytokine profile, and with a particular emphasis on IL-8, for this inflammatory effect is well established in traditionally transplanted lungs [131]. However, little is known regarding the effects of EVLP or cold storage on the lung graft.

In 2011, Sadaria et al. examined the clinical outcomes of EVLP on the lung transplant. The pulmonary function and oxygenation improved upon utilization of EVLP, meaning all lung grafts were suitable for transplantation. The partial pressure of oxygen on 100% fraction of inspired oxygen improved by more than 35% within 2 h of using EVLP compared with donor’s oxygenation. Furthermore, they detected no pulmonary oedema or any histological pathologies. A cytokine profile was taken, and an upregulation of proinflammatory cytokines IL-8, IL-6, and G-CSF were noted [132]. IL-8′s increased expression during EVLP was corroborated in a few other studies [133, 134]. Furthermore, IL-6 is a multifunctional cytokine that has been shown to induce *T*_fh_ cells and consequentially the initiation of germinal centre (GC) formation and induction of B-cells to plasma cells [135].

A pre-clinical study examining the effects of negative pressure ventilation (NPV) on EVLP had resulted in a reduction of inflammation and lung injury [136]. However, as aforementioned, there is no evidence of histological pathologies, despite the increase in proinflammatory cytokines. It is suggested that these mildly expressed cytokines during EVLP are tolerated without any adverse effects. Kakishita et al. had noted a similar effect, whereby an increase in tumour necrosis factor-alpha (TNF-α) and IL-8 were reported, but upon removal of these inflammatory markers by an absorbent membrane, there was no major improvement in pulmonary function [134]. As part of the DEVELOP-UK study, TNF-α and IL-1β were discovered as potential markers of EVLP reconditioning and post-transplant survival. Consequentially, IL-1β therapeutics may decrease the endothelial activation and incidence of graft injury post-transplantation [137].

Cold storage, or rather, the cold ischaemic phase, also results in an increase in proinflammatory markers. An increase in TNF-α, IFN-γ, IL-10 and IL-18 during the ischaemic time whereas they reduced after reperfusion. Conversely, IL-8 had gradually increased over time and reflected a difference in levels between patients who died and who survived; this was not the case in the other inflammatory markers [131]. The neutrophil activator, IL-8, has been shown to underline the development of acute lung injury through the anti-IL-8 autoantibody:IL-8 complexes. These complexes affect neutrophil apoptosis by interacting with the FcγRIIa receptors [138].

### EVLP and cold storage: effects on oxidative stress

An imbalance in favour of free radicals against the neutralising effects of antioxidants results in oxidative stress. Reactive oxygen species (ROS) production is promoted in response to hypoxia and anoxia and the reintroduction of oxygen, where there is a decrease in intracellular ATP and increase in ATP degradation products [139, 140]. However, this process is different from oxidative stress, where there is no decreased ATP and it may occur in cold phase ischaemia during storage [141]. ROS and the oxidative stress response may impact signal transduction and chromatin remodelling and, therefore, regulating the proinflammatory response [142, 143]. This occurs either through the production of lipid peroxidation products or via the direct activation and phosphorylation of stress kinases (JNK, ERK, p38) and redox-sensitive transcription factors NF-kappaB and AP-1. Through this mechanism, ROS is believed to potentially cause allograft injury [144].

Pharmacological reconditioning may impede ischaemia–reperfusion injury. Pre-clinical studies have reflected anti-oxidant properties of Steen solution in EVLP. The solution was able to particularly reduce NADPH oxidase 2 isoform activation, promoting preservation of the lung epithelial cells [145]. Furthermore, 2% sevoflurane administered intravascularly during EVLP has been shown to reduce oxidative stress and the inflammatory response [146]. Hydrogen inhalation during cold storage demonstrated an improvement in oxidative stress indices, including superoxide dismutase activity [147]. Further studies are needed to elucidate the exact effects of EVLP and cold storage on oxidative stress, as this is currently unknown.

Despite the preclinical findings which suggest that EVLP may reduce graft inflammation and oxidative stress, trials such as INSPIRE, NOVEL and HELP have looked into the superiority of one method over another, and reported no short-term survival benefit [122, 148–150], however, one recent study has suggested better lung function at 3 months in the EVLP patients [118]. This is corroborated in a meta-analysis, in which 8 studies involving 1191 patients belong to EVLP and non-EVLP groups were analysed. The EVLP groups had more abnormal donor lung radiographs, and worse donor arterial oxygen tension/inspired oxygen fraction. There were no differences between EVLP and non-EVLP groups in regard to length of postoperative intubation, length of intensive care and hospital stay, postoperative extracorporeal life support/extracorporeal membrane oxygenation use and 30-day or 1-year survival [151]. Ultimately, the purpose of EVLP-treated lung transplant is to allow surgeons to identify potentially viable donor lungs amongst grafts that would otherwise be discarded.

## Conclusion

In this review, we discussed the current global practice surrounding lung transplants, as well as some of the important pre-transplant considerations such as candidate selection, graft ischaemia and graft storage. The recurring theme with all the above considerations is the balance between optimising post-transplant outcomes and minimizing transplant waiting time. With the increasing clinical experience in transplantation and post-transplant care, increasing patients are surviving with grafts which would have been considered suboptimal a few decades ago. Newer technologies such as EVLP may have a role to play in further expanding the donor pool and optimising graft viability, however, this will need to be confirmed through larger sample size studies worldwide.
